# Comparison of knowledge of HIV status and treatment coverage between non-citizens and citizens: Botswana Combination Prevention Project (BCPP)

**DOI:** 10.1371/journal.pone.0221629

**Published:** 2019-08-29

**Authors:** Tafireyi Marukutira, Lisa Block, Mary Grace Alwano, Stephanie Behel, Joseph N. Jarvis, Unoda Chakalisa, Kate Powis, Vladimir Novitsky, William Bapati, Huisheng Wang, Faith Ussery, Refeletswe Lebelonyane, Lisa A. Mills, Janet Moore, Pamela Bachanas

**Affiliations:** 1 Centers for Disease Control and Prevention, Gaborone, Botswana; 2 Public Health, Burnet Institute, Melbourne, Australia; 3 School of Public Health and Preventive Medicine, Monash University, Melbourne, Australia; 4 Northrop Grumman Corporation, Atlanta, Georgia, United States of America; 5 Division of Global HIV/AIDS and TB, Centers for Disease Control and Prevention, Atlanta, Georgia, United States of America; 6 Botswana-UPenn Partnership, Gaborone, Botswana; 7 Department of Clinical Research, Faculty of Infectious and Tropical Diseases, London School of Hygiene and Tropical Medicine, London, United Kingdom; 8 Botswana-Harvard Partnership, Gaborone, Botswana; 9 Harvard T.H Chan School of Public Health, Boston, Massachusetts, United States of America; 10 Tebelopele HIV Testing and Counselling Centre, Gaborone, Botswana; 11 Department of HIV/AIDS Prevention and Care, Botswana Ministry of Health, Gaborone, Botswana; University of the Witwatersrand, SOUTH AFRICA

## Abstract

**Introduction:**

Non-citizens often face barriers to HIV care and treatment. Quantifying knowledge of positive HIV status and antiretroviral therapy (ART) coverage among non-citizens in a high HIV-prevalence country like Botswana that is close to achieving UNAIDS “90-90-90” targets may expose important gaps in achieving universal HIV testing and treatment.

**Methods:**

The Botswana Combination Prevention Project (BCPP) is a pair-matched cluster-randomized trial evaluating the impact of prevention interventions on HIV incidence in 30 rural or peri-urban communities. Community case finding and HIV testing were conducted in home and mobile venues in 15 intervention communities from October 2013-September 2017. In this secondary analysis, we compared HIV positivity, knowledge of positive HIV-status, and ART status among all citizens and non-citizens assessed at intake in the intervention communities.

**Results:**

HIV status was assessed in 57,556 residents in the intervention communities; 4% (n = 2,463) were non-citizens. Five communities accounted for 81% of the total non-citizens assessed. A lower proportion of non-citizens were HIV-positive (15%; n = 369) compared to citizens (21%; n = 11,416) [p = 0.026]; however, a larger proportion of non-citizens did not know their HIV-positive status prior to BCPP testing (75%) as compared to citizens (15%) [p = 0.003]. Among residents with knowledge of their HIV-positive status before BCPP, 79% of the non-citizens (72/91) were on ART compared to 86% (8,267/9,652) of citizens (p = 0.137).

**Conclusions:**

Although non-citizens were less likely to know their HIV-positive status compared to citizens, there were no differences in treatment uptake among non-citizens and citizens who knew their status. Designing interventions for non-citizens that provide HIV testing and treatment services commensurate to that of citizens as well as targeting communities with the largest number of non-citizens may help close a meaningful gap in the HIV care cascade and ensure ethical treatment for all HIV-positive persons.

**Trial registration:**

ClinicalTrials.gov: NCT01965470 (Botswana Combination Prevention Project).

## Introduction

Knowing one’s positive HIV status and initiating and staying on treatment are essential for combatting the HIV epidemic. The UNAIDS “90-90-90” targets establish goals for 90% of all HIV-infected persons to know their status, 90% of persons with diagnosed HIV infection to receive sustained antiretroviral therapy (ART), and 90% of all persons receiving ART to be virally suppressed by 2020 [1]. Achieving the “90-90-90” targets and scaling up to “95-95-95” by 2030 is projected to reduce HIV incidence and mortality by 90% compared to 2010 levels [2].

Botswana, a high HIV prevalence country with 22.8% of its adult population infected with the HIV virus [3], is currently on target to reach the “90-90-90” targets [3, 4]. With strong political will and leadership, Botswana was one of the first countries with a generalized HIV epidemic to commit to a national HIV treatment program and one of the first African countries to offer free HIV testing and adopt a universal treatment policy regardless of CD4+ T-cell count [5]. The ART program, in place since 2002, provides free HIV care and treatment services to Botswana citizens. Despite the great strides that the country has made in the HIV epidemic response, incident cases are still being reported in the country [6, 7]. Little research has been conducted in Botswana to determine if coverage of the HIV treatment cascade extends across sub-populations who may have less access to health care.

One subpopulation in Botswana who may not be adequately covered by the HIV care cascade is the non-citizen population that is currently residing in the country. Migratory sub-populations may experience poor health outcomes and contribute to the ongoing epidemic because of reduced access to HIV testing and treatment programs designed to serve their unique needs [8]. Botswana attracts migrant workers and shares close proximity to bordering countries (South Africa, Zambia and Zimbabwe) with high HIV prevalence [4]. In 2015, it was estimated that there were 161,000 non-citizens in Botswana representing about 7% of the total population of 2.3 million people [9]. Current Botswana policy offers free health services and ART to all citizens and spouses of citizens but requires payment for these same services from non-citizens who are currently living and working in the country.

The largest cities in Botswana attracting migrant labor are geographically close to the aforementioned neighboring countries with high HIV prevalence. Migrant workers becoming infected upon reaching Botswana may be less willing to access HIV testing given that they are not eligible for free treatment. In addition, HIV-positive non-citizens receiving treatment in their country of origin may be unlikely to return for prescription refills and ongoing health care and, thus, are at risk for developing drug resistance. Consequently, Botswana may have a substantial group of people not receiving WHO and UNAIDS recommended universal HIV testing and treatment to reduce mortality among infected persons and to promote epidemic control.

There are limited data on the extent of HIV infection within the non-citizen population in Botswana [10], and the extent to which HIV-positive persons are receiving essential HIV-related health services. Through a sub-analysis we sought to quantify differences in HIV-positivity, HIV testing and knowledge of status, and ART coverage between citizens and non-citizens as a means to further understand the implications for reaching the 90-90-90 targets and HIV epidemic control in Botswana.

## Methods

### Design

The Botswana Combination Prevention Project (BCPP) is a cluster-randomized HIV prevention trial evaluating the impact of a package of prevention interventions on population-level HIV incidence in 30 rural or peri-urban communities in Botswana with an average population of 6,000 per community. Fifteen communities were randomly assigned to the intervention arm and 15 to the arm receiving standard of care services. A full description of the study design is available elsewhere [11]. HIV and ART status were assessed among residents in the 15 intervention communities from October 2013-September 2017. Secondary analyses of data collected at intake from the intervention communities only are presented here. Information on non-citizens was not collected in the 15 control communities.

### Participants

All community residents 16–64 years old were interviewed and assessed for HIV status after providing verbal consent as per Botswana’s HIV testing guidelines for anyone aged ≥16 years. [12]. Community residency was established through self-report and defined as spending at least three nights per month in the community. Persons were classified as citizens if they had an Omang (national identifier) or Botswana passport. Non-citizenship was self-reported, and passport numbers were collected if available. All persons were asked if they had ever tested HIV-positive. Those who knew their HIV-positive status were asked for documentation of HIV status. Persons who did not know their HIV status, who did not have documentation of an HIV-positive status, or who did not have documentation of a negative HIV test within the preceding three months were offered HIV testing. All HIV testing and data collection were conducted by trained local lay counselors who were fluent in Setswana, English and other local languages.

### Setting and procedures

Community campaigns composed of both home and mobile testing strategies were employed to reach all community residents aged 16–64 years. For the home-based strategy, counselors received lists of all household plots for each of the intervention communities (obtained from Google maps and Botswana 2011 census data). GPS coordinates were used to locate the households on handheld tablets. Counselors returned to households up to three times in order to find residents at home for assessment of HIV status and HIV testing if indicated. The study teams conducted evening and weekend HIV testing and made appointments for home-based testing for times when absent residents would be home. Mobile testing was made available throughout communities at high traffic locations including markets and transport hubs. In addition, mobile testing was provided at community events, health fairs, work sites and shebeens/bars. Persons accessing mobile venues completed the same study procedures as persons participating in the home. All HIV infected persons not on ART identified in home or mobile venues were counseled on the benefits and importance of early care and treatment for their health and the health of partners and children.

### Data collection, variable measurement, and analyses

#### Data collection

All responses to structured interview questions and results of HIV tests were collected on encrypted handheld tablets, which were synchronized daily with the research database and removed from the tablet after synchronization to maximize data confidentiality.

For field-based rapid HIV testing, finger stick testing using Unigold (Trinity Biotech, Wicklow, Ireland) and KHB (Shanghai Kehua Bio-engineering Co Ltd, Shanghai, China) tests was conducted following the Botswana national testing algorithm. Quality control was assured by re-testing samples from each community in the reference laboratory using enzyme-linked immunosorbent assay (ELISA) and HIV-1 Western Blot testing.

#### Measurement of study variables

Algorithms were designed to measure three main outcomes: HIV positivity, knowledge of positive HIV status prior to BCPP, and ART coverage.

1. HIV-positive status was defined as a positive result on an HIV test conducted through BCPP or documentation of HIV positivity at intake (i.e., previous positive test result, health card, ART pill bottle, or electronic medical record) of unique individuals found in either home or mobile venues.
2. Knowledge of positive HIV status prior to BCPP intake was defined as the show of documentation of HIV positivity prior to BCPP interview date (i.e., test result, health card, ART pill bottle, or electronic medical record).
3. Current on ART was defined by documentation of current use of ART (health cards, prescriptions, pill bottles, electronic medical records) at the time of BCPP intake.

#### Analysis

SAS 9.4 was used for all statistical analyses. SAS PROC SURVEYFREQ was used to produce frequencies adjusting for clustering of observations within communities. Bivariate tests of association were conducted using Rao-Scott Chi-square tests, with a critical alpha level of 0.05. SAS PROC SURVEYLOGISTIC was used to test for association between sociodemographic factors of interest and HIV positivity, prior knowledge of HIV-positive status and ART status. These models produced unadjusted and adjusted odds ratios (ORs) and 95% confidence limits.

#### Ethics approval

The study was approved by the Centers for Disease Control and Prevention Institutional Review Board (Protocol #6475) and the Botswana Health Research and Development Committee (Institutional Review Board of the Botswana Ministry of Health and Wellness). The study was monitored by an Independent Data and Safety Monitoring Board.

## Results

### Characteristics of persons assessed for HIV status

BCPP interviewed 60,139 residents in the intervention communities. Among those interviewed, HIV status was ascertained in 96% (n = 57,556) of residents (53% female, 47% male) with only 4% refusing to be tested or show documentation of their HIV status. Overall, 4% (n = 2463) of the assessed residents self-reported as non-citizens. The refusal rate among non-citizens was higher than the refusal rate among citizens (6% vs. 4%; p = 0.028).

Table 1 displays characteristics comparing non-citizens to citizens. A higher proportion of non-citizens were male (64%) compared to the proportion of citizens (46%; p = 0.001). The average age among non-citizens was slightly younger compared to citizens with the greatest proportion of non-citizens falling between the ages of 25–34 and the greatest proportion of citizens falling between the ages of 35–64 (p = 0.002). Non-citizens were more likely to be assessed in mobile venues (65%) compared to citizens (51%; p = 0.009).

**Table 1:** Demographic characteristics and testing venues of individuals assessed for HIV status.

Variable	Non-citizens (N = 2,463) n (%)	Citizens (N = 55,093) n (%)	*P*-value*
**Sex**			**<0.001**
Female	885 (36)	29,802 (54)	
Male	1,578 (64)	25,291 (46)	
**Age (in years)**			**0.002**
16–24	606 (25)	17,360 (32)	
25–34	1,059 (43)	16,344 (30)	
35–64	798 (32)	21,389 (39)	
**Testing Venue**			**0.009**
Assessed in Home	851 (35)	27,201 (49)	
Assessed in Mobile unit	1,612 (65)	27,892 (51)	

Values are presented as frequency (%).

* p value calculated using the Rao-Scott modified Chi square test

### HIV-positive status

Among all residents assessed, 21% (n = 11,785) were HIV-positive. Non-citizens represented 3% (n = 369) of all HIV-positives identified. Five of the 15 communities (Masunga, Mathangwane, Nkange, Oodi and Tati Siding) accounted for 83% of the HIV-positive non-citizens identified. Four out of five of communities were located just outside of Francistown, the second largest city in Botswana, which is close to the border of Zimbabwe. The proportion of residents who were HIV-positive ranged from 9% to 28% across communities among non-citizens and 14% to 29% among citizens.

A smaller proportion of non-citizens were HIV-positive (15%; n = 369) as compared to citizens (21%; n = 11,416) [p = 0.026]. Table 2 presents bivariate analyses of characteristics of persons who were HIV-positive among those assessed. A smaller proportion of the HIV-infected non-citizens were female compared to citizens (48% vs. 66%; p = 0.001). A significantly higher proportion of HIV-positive non-citizens (46%) were identified in mobile venues as compared to citizens (22%) [p = 0.016]. In a multivariate analysis, citizenship remained associated with HIV positivity; non-citizens were 20% less likely to be HIV infected compared to citizens after controlling for age, sex, and peri-urban vs. rural community of residence (Table 3).

**Table 2:** Characteristics of HIV-Positive Non-Citizens and Citizens.

Variable	HIV+ Non-citizens (N = 369) n (%)	HIV+ Citizens (N = 11,416) n (%)	*P-value* *
**Sex**			**0.001**
Female	176 (48)	7,586 (66)	
Male	193 (52)	3,830 (34)	
**Age (in years)**			**<0.001**
16–24	43 (12)	965 (8)	
25–34	145 (39)	2,702 (24)	
35–64	181 (49)	7,749 (68)	
**Testing venue**			**0.016**
HIV+ in Home	198 (54)	8,958 (78)	
HIV+ in Mobile unit	171 (46)	2,458 (22)	

Values are presented as frequency (%).

* P-value calculated using the Rao-Scott modified Chi square test.

**Table 3:** Association of sociodemographic variables with HIV infection, n = 57,556.

Variable	Unadjusted OR (95% CI)	p	Adjusted OR (95% CI)	p
**Sex**				
Female	1.92 (1.80, 2.06)	<0.001*	1.98 (1.83, 2.13)	<0.001*
Male	ref	---	ref	---
**Age**				
16–24	0.11 (0.09, 0.12)	<0.001*	0.11 (0.09, 0.12)	<0.001*
25–34	0.35 (0.31, 0.39)	<0.001*	0.36 (0.32, 0.40)	<0.001*
35–49	ref	---	ref	---
**Citizenship**				
Non-citizen	0.68 (0.55, 0.83)	0.001*	0.80 (0.66, 0.96)	0.02*
Citizen of Botswana	ref	---	ref	---
**Community residency**				
Peri-urban	0.86 (0.63, 1.19)	0.34	0.93 (0.67, 1.27)	0.61
Rural	ref	---	ref	---

*Significant at α = .05

### Prior knowledge of HIV status

Of the 11,785 HIV positive persons identified, 83% (n = 9,743) had documentation of being HIV-positive and 17% (n = 2,042) tested HIV-positive in BCPP. A significantly smaller proportion of non-citizens knew their HIV-positive status prior to BCPP testing (25%) than did citizens (85%; p = 0.003). Table 4 displays the multivariate analysis of selected sociodemographic factors and prior knowledge of HIV-positive status. Non-citizens were 93% less likely to know their HIV positive status at intake compared to citizens adjusting for sex, age, and rural vs. peri-urban community (p<0.001).

**Table 4:** Association of sociodemographic variables with prior knowledge of HIV-positive status among those assessed as HIV-positive, n = 11,785.

Variable	Unadjusted OR (95% CI)	p	Adjusted OR (95% CI)	p
**Sex**				
Female	2.25 (2.06, 2.45)	<0.001*	2.72 (2.46, 2.99)	<0.001*
Male	ref	---	ref	---
**Age**				
16–24	0.24 (0.19, 0.29)	<0.001*	0.19 (0.15, 0.24)	<0.001*
25–34	0.40 (0.35, 0.46)	<0.001*	0.36 (0.32, 0.40)	<0.001*
35–49	ref	---	ref	---
**Citizenship**				
Non-citizen	0.06 (0.04, 0.08)	<0.001*	0.07 (0.05, 0.10)	<0.001*
Citizen of Botswana	ref	---	ref	---
**Community residency**				
Peri-urban	0.64 (0.42, 0.97)	0.04	0.78 (0.51, 1.18)	0.22
Rural	ref	---	ref	---

*Significant at α = .05

### Currently on ART

Among all HIV-positives, 71% (8,339/11,785) were currently on ART. Among HIV-positive non-citizens who knew their status prior to BCPP, 79% (72/91) were on ART compared to 86% (8,267/9,652) of citizens with prior knowledge of their HIV status (p = 0.137). Table 5 displays the multivariate analysis between selected sociodemographic factors and ART status among those with prior knowledge of their HIV-positivity. Citizenship was not related to being on ART among persons who knew their positive status (p = 0.38).

**Table 5:** Association of sociodemographic variables to ART use among persons who had prior knowledge of HIV-positive status, n = 9,743.

Variable	Unadjusted OR (95% CI)	p	Adjusted OR (95% CI)	p
**Sex**				
Female	0.97 (0.86, 1.09)	0.56	1.13 (1.00, 1.27)	0.05
Male	ref	---	ref	---
**Age**				
16–24	0.42 (0.35, 0.51)	<0.001*	0.42 (0.35, 0.50)	<0.001*
25–34	0.43 (0.38, 0.48)	<0.001*	0.42 (0.37, 0.48)	<0.001*
35–49	ref	---	ref	---
**Citizenship**				
Non-citizen	0.63 (0.31, 1.30)	0.19	0.75 (0.38, 1.48)	0.38
Citizen of Botswana	ref	---	ref	---
**Community residency**				
Peri-urban	0.74 (0.56, 0.97)	0.03*	0.75 (0.57, 0.98)	0.04*
Rural	ref	---	ref	---

*Significant at α = .05

## Discussion

The results presented in this paper offer insight into the HIV epidemic among non-citizen residents of Botswana and the gaps in coverage of vital HIV services for individual health and epidemic control. Among residents assessed, HIV positivity among non-citizens was 15%. Only 25% of HIV-positive non-citizens knew their HIV status and thus the vast majority of non-citizens were not receiving treatment. Among non-citizens who knew their positive status, 79% were receiving ART, a percentage similar to ART use among citizens (86%). In sum, these findings suggest that this important sub-population in Botswana has substantial need for HIV services but is critically underserved.

Data from extensive community-based HIV case finding and testing services showed that non-citizens accounted for 4% of the overall population in the 15 intervention communities. However, five communities accounted for 81% of the non-citizens assessed and 83% of the positive non-citizens identified. These findings suggest that HIV interventions for this sub-population should be targeted to specific geographic locations which are most likely to attract migrant labor and/or refugees.

Botswana offers free HIV testing to non-citizens, but at BCPP intake only 25% of non-citizens knew they were HIV-positive compared to 85% of citizens. Non-citizens may not proactively seek HIV testing for a myriad of reasons related to their immigration status including disrupted living situations, lack of support from family members, negative attitudes of healthcare workers, fear of deportation, stigma, and lack of availability of HIV treatment even if they know their status. Evidence in other studies have shown similar factors to be challenges for immigrants accessing healthcare in Southern Africa [13,14].

Despite the low level of knowledge of HIV status at intake, 94% percent of non-citizens offered an HIV test consented to testing, and many sought testing services in mobile venues. More non-citizens were assessed in mobile venues than in the home, but more HIV-positive non-citizens were identified in the home. These findings suggest that non-citizens are receptive to HIV testing, and both home and mobile testing services reach a high proportion of non-citizens so should be part of the testing strategy for reaching this group.

Unlike the HIV-positive citizens, the majority of HIV-positive non-citizens were not on ART when first encountered by BCPP. Treatment options are likely cost-prohibitive for many within this group and they are not eligible for government-subsidized ART. Thus, the health of HIV-positive non-citizens is probably greatly compromised and viral suppression coverage in the community as a whole is unattainable. If non-citizens access ART in their countries of origin and experience treatment interruptions due to cost when they migrate, this may introduce drug mutations with implications if the resistant virus is further transmitted.

In the Southern African Development Community (SADC) that includes Botswana, there are concerns that the HIV response in the region does not adequately address the movement of healthcare users within and between countries thereby impacting universal test and treat programs[13]. In a region most impacted by HIV, comprehensive, sustainable, and inclusive programs are required. Addressing potential pockets of undiagnosed HIV in subpopulations may improve health outcomes of these groups and reduce continued occurrence of new HIV infections.

Several methodological limitations existed within the study. Measuring the non-citizens’ knowledge of positive HIV status prior to the BCPP intervention was limited to the individual’s ability to produce documentation of a prior HIV test result. Without the government-issued Omang, the study team was unable to obtain any clinical data on HIV testing and ART status for non-citizens using a unique identifier as was possible with the citizen group. Clinical records (e.g., HIV test results occurring before the interview date, ART initiation dates prior to the interview, etc.) were used to augment the documentation of intake data among the citizens but unavailable for the non-citizens. Therefore, it is possible that a higher proportion of non-citizens were “known HIV-positive” at intake than reported. Another methodological limitation to the study was that BCPP was conducted in rural and peri-urban communities and not the larger cities which serve as economic hubs and typically draw immigrants seeking work in Botswana. Therefore, rural/peri-urban communities may not be representative of where most non-citizens reside in Botswana. This design feature could explain the difference between the estimated 7% non-citizen population in Botswana and BCPP’s report of 4% non-citizenship among all community residents assessed for HIV. Thus, the HIV positivity and treatment rates found among non-citizens may or may not be generalizable to the country as a whole.

## Conclusions

Non-citizens have lower levels of knowledge of positive HIV status compared to citizens; thus, overall ART coverage is low in this sub-population in Botswana. When they know their HIV-positive status, rates of ART uptake are similar among citizens and non-citizens. In a country close to achieving their “90-90-90” goals but still reporting new HIV infections, it is imperative to examine gaps in the HIV care cascade at a sub-population level. Increasing access to HIV care by eliminating social and monetary barriers for non-citizens may improve health outcomes for all and possibly contribute to epidemic control in Botswana. Focusing intervention efforts on the geographic locations where most non-citizens reside may produce the greatest benefit.

## Supporting information

S1 File Data use statement. (PDF)

S2 File SAS Program code. (PDF)

S3 File Dataset. (CSV)

S1 Table Data dictionary. (PDF)
