# Supplementary material for: Comparison of knowledge of HIV status and treatment coverage between non-citizens and citizens: Botswana Combination Prevention Project (BCPP)
Source: PLoS One. 2019 Aug 29;14(8):e0221629. doi: 10.1371/journal.pone.0221629 (PMC6715216; doi:10.1371/journal.pone.0221629)
Supplement: S1 File — (PDF) [file pone.0221629.s001.pdf]

Data Use Statement
De-Identified Data

Background

In support of its mission, the Centers for Disease Control and Prevention (CDC) collects, generates, stores, uses, and routinely provides access to public health data. Public health and scientific advancement are best served when public health data are released to or shared with other public health agencies, academic researchers, private researchers (if appropriate), and other partners in an open, timely, and appropriate way. Pursuant to its mission, the CDC *Policy on Public Health and Nonresearch Data Management and Access, January 26, 2016* seeks to make accessible public health data the agency has collected and generated subject to limits imposed by law, ethical considerations, resources, technology, data quality, and protection of data from physical and electronic risks to privacy and confidentiality.

Data and Documentation for Release

The data set "BCPP_NonCitizen_vs_Citizen" and associated documentation are being released in compliance with the Policy on Public Health Research and Nonresearch Data Management and Access, January 26, 2016, as Public Access and subject to the following provisions.

These data were collected with the support of the President's Emergency Plan for AIDS Relief (PEPFAR) through the Centers for Disease Control and Prevention (CDC) under the terms of Cooperative Agreements U2G GH000073 and U2G GH000419.

Provisions

By use of this data set, the user acknowledges and agrees to the following conditions:

- No attempt will be made to identify records contained in the data provided under this Data Use Statement or to link these data with other data sources for identification purposes.
- Licensing – this data set and documentation are available under the Creative Commons Attribution license (CC BY) https://creativecommons.org/licenses/by/4.0/.

Under the terms of this license, you are allowed to download the data and may share them with others as long as appropriate credit is given (*Attribution*) but not in a manner that suggests that CDC endorses the use of the data. If you want to use this data without giving credit to CDC or for endorsement purposes, permission must be sought first from CDC.

Recommended Citation

Botswana Combination Prevention Project. Botswana_NonCitizen_vs_Citizen. <*INSERT RELEASE DATE: dd mon yyyy*>. Available from: <*INSERT* URL>.

General Disclaimer

Every effort has been made to provide accurate and complete information contained in this data set and documentation. However, we cannot guarantee that there will be no errors. CDC does not assume any legal liability for the accuracy, completeness, or usefulness of any information, product, or process disclosed herein, or represents that use of such information, product, or process would not infringe on privately owned rights.
