# Supplementary material for: Comparison of knowledge of HIV status and treatment coverage between non-citizens and citizens: Botswana Combination Prevention Project (BCPP)
Source: PLoS One. 2019 Aug 29;14(8):e0221629. doi: 10.1371/journal.pone.0221629 (PMC6715216; doi:10.1371/journal.pone.0221629)
Supplement: S2 File — (PDF) [file pone.0221629.s002.pdf]

```
*********************************************************************************
* This SAS program may be used to reproduce tables 1-6 in the manuscript titled  *
* "Comparison of knowledge of HIV status and treatment coverage between citizens *
* and non-citizens: Botswana Combination Prevention Project (BCPP)"             *
*********************************************************************************;

dm 'clear output; clear log'; options pageno=1;

*import csv data and create a temp sas dataset named, noncitizen;
data work.noncitizen;
 %let _EFIERR_ = 0; /* set the ERROR detection macro variable */
infile '\\cdc.gov\project\CGH_DGHA_BCPP_Data\_Clearance\_Manuscript Data Release
Clearance\1_NonCitizen\Resubmit\bcpp_noncitizen_vs_citizen_v2.csv' delimiter = ',' MISSOVER
DSD lrecl=32767
 firstobs=2 ;
informat participantid best32. community $15. area $15. gender $15. citizenshipstatus $15.  agecat3 $15.
             testvenue $15. hivstatus $15. hivsposknow $15. onartattest $15. assessgroup $15. testyieldgroup $15. ;
format participantid best12. community $15. area $15. gender $15. citizenshipstatus $15. agecat3 $15.
          testvenue $15. hivstatus $15. hivsposknow $15. onartattest $15. assessgroup $15. testyieldgroup $15. ;
input participantid community $ area $ gender $ citizenshipstatus $ agecat3 $
         testvenue $ hivstatus $ hivsposknow $ onartattest $ assessgroup $ testyieldgroup $;
if _ERROR_ then call symputx('_EFIERR_',1);  /* set ERROR detection macro variable */
 run;

*** Table 1 ***;
 title 'BCPP Non-Citizen vs. Citizen Public Release Data';
title2 'Table 1:  Demographics by Citizenship Status';
proc freq data = noncitizen;
table (gender agecat3 testvenue)*citizenshipstatus / norow nopercent;
where assessgroup = "Yes";
run;

proc surveyfreq data = noncitizen;
table (gender agecat3 testvenue)*citizenshipstatus / chisq1;
cluster community;
where assessgroup = "Yes";
run;

*** Table 2 ***;
title 'BCPP Non-Citizen vs. Citizen Public Release Data';
title2 'Table HIV-Positivity among non-citizens by community';
proc freq data = noncitizen;
table (community)*hivstatus / nocol nopercent;
where assessgroup = "Yes" and citizenshipstatus = "Noncitizen";
run;

*** Table 3 ***;
title 'BCPP Non-Citizen vs. Citizen Public Release Data';
title2 'Table 3:  Characteristics of HIV-Positive Non-Citizens and Citizens';
proc freq data = noncitizen;
```

```
table (gender agecat3 testvenue)*citizenshipstatus / norow nopercent;
where assessgroup = "Yes" and hivstatus = "HIV+";
run;

proc surveyfreq data = noncitizen;
table (gender agecat3 testvenue)*citizenshipstatus / chisq1;
where assessgroup = "Yes" and hivstatus = "HIV+";
cluster community;
run;

*** Table 4 ***;
title 'BCPP Non-Citizen vs. Citizen Public Release Data';
title2 'Table 4:  Association of sociodemographic variables with HIV infection';
title3 'Unadjusted Models';
%macro varlist (var=,reflevel=);
proc surveylogistic data=noncitizen;
class
        &var. (ref=&reflevel.)
  / param = ref;
model hivstatus (event='HIV+') = &var.;
cluster community;
where assessgroup = "Yes";
run;
%mend;
%varlist(var=gender, reflevel='Male');
%varlist(var=agecat3, reflevel='35-64');
%varlist(var=citizenshipstatus, reflevel='Citizen');
%varlist(var=area, reflevel='Rural');

title 'BCPP Non-Citizen vs. Citizen Public Release Data';
title2 'Table 4:  Association of sociodemographic variables with HIV infection';
title3 'Full Model';
proc surveylogistic data=noncitizen;
class
  gender (ref='Male')
  agecat3 (ref='35-64')
  citizenshipstatus (ref='Citizen')
  area (ref='Rural')
 / param = ref;
model hivstatus (event='HIV+') = gender agecat3 citizenshipstatus area;
cluster community;
where assessgroup = "Yes";
run;

*** Table 5 ***;
title 'BCPP Non-Citizen vs. Citizen Public Release Data';
title2 'Table 5:  Association of sociodemographic variables with prior knowledge of HIV-positive status';
title3 'among those assessed as HIV-positive';
title4 'Unadjusted Models';
%macro varlist (var=,reflevel=);
```

```
proc surveylogistic data=noncitizen;
class
        &var. (ref=&reflevel.)
  / param = ref;
model hivsposknow (event='Known HIV+') = &var.;
cluster community;
where assessgroup = "Yes" and hivstatus = "HIV+";
run;
%mend;
%varlist(var=gender, reflevel='Male');
%varlist(var=agecat3, reflevel='35-64');
%varlist(var=citizenshipstatus, reflevel='Citizen');
%varlist(var=area, reflevel='Rural');

title 'BCPP Non-Citizen vs. Citizen Public Release Data';
title2 'Table 5:  Association of sociodemographic variables with prior knowledge of HIV-positive status';
title3 'among those assessed as HIV-positive';
title4 'Full Model';
proc surveylogistic data=noncitizen;
class
  gender (ref='Male')
  agecat3 (ref='35-64')
  citizenshipstatus (ref='Citizen')
  area (ref='Rural')
 / param = ref;
model hivsposknow (event='Known HIV+') = gender agecat3 citizenshipstatus area;
cluster community;
where assessgroup = "Yes" and hivstatus = "HIV+";
run;

*** Table 6 ***;
title 'BCPP Non-Citizen vs. Citizen Public Release Data';
title2 'Table 6:  Association of sociodemographic variables to ART status';
title3 'among persons who had prior knowledge of HIV-positive status';
title4 'Unadjusted Models';
%macro varlist (var=,reflevel=);
proc surveylogistic data=noncitizen;
class
        &var. (ref=&reflevel.)
  / param = ref;
model onartattest (event='Yes') = &var.;
cluster community;
where assessgroup = "Yes" and hivstatus = "HIV+" and hivsposknow = "Known HIV+";
run;
%mend;
%varlist(var=gender, reflevel='Male');
%varlist(var=agecat3, reflevel='35-64');
%varlist(var=citizenshipstatus, reflevel='Citizen');
%varlist(var=area, reflevel='Rural');
```

```
title 'BCPP Non-Citizen vs. Citizen Public Release Data';
title2 'Table 6:  Association of sociodemographic variables with prior knowledge of HIV-positive status';
title3 'among persons who had prior knowledge of HIV-positive status';
title4 'Full Model';
proc surveylogistic data=noncitizen;
class
  gender (ref='Male')
  agecat3 (ref='35-64')
  citizenshipstatus (ref='Citizen')
  area (ref='Rural')
 / param = ref;
model onartattest (event='Yes') = gender agecat3 citizenshipstatus area;
cluster community;
where assessgroup = "Yes" and hivstatus = "HIV+" and hivsposknow = "Known HIV+";
run;
```
