# Supplementary material for: Comparison of knowledge of HIV status and treatment coverage between non-citizens and citizens: Botswana Combination Prevention Project (BCPP)
Source: PLoS One. 2019 Aug 29;14(8):e0221629. doi: 10.1371/journal.pone.0221629 (PMC6715216; doi:10.1371/journal.pone.0221629)
Supplement: S1 Table — (PDF) [file pone.0221629.s004.pdf]

**Study Name**	Botswana Combination Prevention Project		
**Study Country**	Botswana		
**Protocol URL**	https://clinicaltrials.gov/ct2/show/NCT01965470?cond=HIV&cntry=BW&draw=2&rank=14		
**Variable Name**	**Variable Description**	**Response**	**Count**
participantid	Participant ID		60139
community	Intake community	Community1	2173
		Community2	5848
		Community3	2937
		Community4	3468
		Community5	4388
		Community6	4177
		Community7	3213
		Community8	2518
		Community9	4002
		Community10	1950
		Community11	5858
		Community12	4099
		Community13	3119
		Community14	6079
		Community15	6310
area	Community location	Rural	36,657
		Peri-urban	23,482
gender	Gender	Female	31939
		Male	28200
citizenshipstatus	Citizenship status at intake	Citizen	57506

		Noncitizen	2633
agecat3	Age at intake	16-24	18598
		25-34	18303
		35-64	23238
testvenue	Intake test venue/location	Home	30424
		Mobile	29715
hivstatus	HIV status at intake	HIV-	45771
		HIV+	11785
		Refused	2583
hivsposknow	Knowledge of HIV positive status at intake	Known HIV+	9743
		NA	48354
		Newly identified HIV+	2042
onartattest	HIV-positive who had documentation of being on ART at the time of intake and HIV assessment	NA	48354
		No	3446
		Yes	8339
assessgroup	Flag to subset to participants for HIV assessment analysis	No	2583
		Yes	57556
